# Ecological Psychology and Enactivism: A Normative Way Out From Ontological Dilemmas

**DOI:** 10.3389/fpsyg.2020.01637

**Published:** 2020-07-30

**Authors:** Manuel de Pinedo García

**Affiliations:** Departamento de Filosofía I, Universidad de Granada, Granada, Spain

**Keywords:** enactivism, ecological psychology, affordances, normativity, dispositionalism, analytic philosophy, representationalism, agency

## Abstract

Two important issues of recent discussion in the philosophy of biology and of the cognitive sciences have been the ontological status of living, cognitive agents and whether cognition and action have a normative character *per se*. In this paper I will explore the following conditional in relation with both the notion of affordance and the idea of the living as self-creation: if we recognize the need to use normative vocabulary to make sense of life in general, we are better off avoiding taking sides on the ontological discussion between eliminativists, reductionists and emergentists. Looking at life through normative lenses is, at the very least, in tension with any kind of realism that aims at prediction and control. I will argue that this is so for two separate reasons. On the one hand, understanding the realm of biology in purely factualist, realist terms means to dispossess it of its dignity: there is more to life than something that we simply aim to manipulate to our own material convenience. On the other hand, a descriptivist view that is committed to the existence of biological and mental facts that are fully independent of our understanding of nature may be an invitation to make our ethical and normative judgments dependent on the discovery of such alleged facts, something I diagnose as a form of representationalism. This runs counter what I take to be a central democratic ideal: while there are experts whose opinion could be considered the last word on purely factual matters, where value is concerned, there are no technocratic experts above the rest of us. I will rely on the ideas of some central figures of early analytic philosophy that, perhaps due to the reductionistic and eliminativist tendencies of contemporary philosophy of mind, have not been sufficiently discussed within post-cognitivist debates.

## Introduction

I will begin by distinguishing between two forms of antirepresentationalism, one regarding cognition and the other regarding language. I will then claim that, even after having presented a serious challenge and alternatives to the former, there can be a residual form of linguistic representationalism behind the thought that we are in the business of describing facts and referring to free-standing properties when we speak of affordances or give normative explanations of agency (section “Ontological Approaches to Affordances and Normativity”). In section “Non-descriptivism and Rule-Following” I will review some alternatives to descriptivism that draw from early and recent analytic philosophy: some vocabularies, normative, evaluative, intentional, are fundamental for our understanding of nature but they do not bring commitments to additional entities. I will then explore how these ideas apply to the possibility of non-social normativity (section “Non-social Normativity”) and to the relationship between ontological and normative/ethical perspectives on life, cognition and agency (section “Ontology and Ethics”). My central target is to free up a space after exposing three apparent ontological dilemmas as leading to dead ends: either affordances are intrinsic properties or there is no distinction between describing and evaluating (section “Ontological Approaches to Affordances and Normativity”); either the mind has a causal role in nature or we should abandon our mental vocabulary; and (section “Non-descriptivism and Rule-Following”) either values and norms exist independently of evaluative practices or they are a mere projection from a provisional stance (section “Ontology and Ethics”).

The present special issue deals with the convergence and complementarity between enactivism and ecological psychology. This is a refreshing and much needed alternative to one common approach to the relationship between both research programs, namely trying to highlight the alleged superiority of one over the other regarding, say, learning, the role of the agent, the role of the environment or the possibility of giving explanations that scale up to linguistic or social phenomena (Varela et al., 1991; Flament-Fultot et al., 2016; Di Paolo et al., 2017). The topic can be an invitation for an alternative take on the issue, which I won’t pursue, but I assume that is both feasible and desirable: to embark on an ecumenical collaboration between ecological psychology and enactivism, putting together the strengths of one and the other for the major glory of a positive alternative to representationalism and cognitivism (Heras-Escribano, 2019a). However, I think that there is a less explored perspective regarding the convergence of both traditions, one that precisely aims at deepening the antirepresentionalist character of the new paradigm by warning against a common danger that lies ahead, in slightly different forms, for enactivism and ecological psychology, the danger of conflating normative questions with ontological, descriptive ones.

To do so I’ll draw on a philosophical tradition, analytic philosophy, which is often ignored in the post-cognitivist discussions in favor of phenomenology, perhaps because many of its main representatives in the second half of the last century have embraced physicalist or functionalist agendas in the philosophy of mind. However, I think that such agendas are the result of deliberately misunderstanding or plainly ignoring the ideas of analytic philosophers from the previous generation. In which way can a philosophical tradition centered on conceptual analysis often performed without much attention to empirical issues illuminate debates regarding, as in our case, the relation between ecological and enactive approaches to cognition? I think that, at the very least, some methodological clarity may be obtained when thinking about some questions that have been a source of perplexity for philosophy through the centuries—in the case at hand, questions such as “what is life” or “what is mind” or “what is agency”—in terms of the kind of thing that do we do when we say that something is alive or minded or an agent. The questions may become more tractable as well as less abstract and more related to practice.

I can be illuminating to call attention to the fact that the two above-mentioned philosophical strands, phenomenology and analytic heterodox, together with pragmatism, constitute the core of Rorty (1979). The reason I believe it is important to briefly go back to Rorty is that his opposition to representationalism has two sides that are not always distinguished, a cognitive/epistemic one and a linguistic one: we can pursue a conception of the mind with no representations mediating between the cognitive agent and the world and still retain the idea that the main function of language is to represent reality, to describe facts, objects and properties. The mirror of nature that Rorty wants to expel from philosophy is both the mind as a mirror and language as a mirror. While a lot of attention has been paid to the problems of representational understandings of the mind within the cognitive sciences, and both ecological psychology and enactivism can be seen as deeply articulated positive non-representational alternatives (both avoiding the idea that cognition is computation over internal or external representations), antirepresentationalist views of language have tended to remain within the confines of traditional, purely conceptual analytic philosophy. Post-cognitivism can benefit from an emphasis on the idea that some, perhaps most, of our linguistic practices don’t aim to describe a reality that is there anyway but to take an evaluative stance. To think of affordances as intrinsic properties of things that exist independently of the agents that could perceive and take advantage of them and to think of normativity as a describable feature of the pair agent/environment may be two sides of the same conceptual pitfall.

## Ontological Approaches to Affordances and Normativity

As I mentioned above, I am highly optimistic regarding the complementation between ecological psychology and enactivism toward the common project of replacing a representationalist, intellectualist and computationalist conception of life and cognition. I think that there would be a lot to be gained if the different emphases on the meaning-making and evaluative character of cognitive agents, on the one hand, and on the direct perception of information in the environment that is relevant for the agent, on the other, are just that, a difference of emphasis. The plea that I hope to make for keeping ontology and normativity apart is grounded on the rejection of: (1) the realist idea that values—meanings, relevancies, affordances—could be individuated without any reference to practices of evaluation (either those of the agent that finds them of value or our own normative explanations of such interactions) and (2) what we could call a purely projectivist understanding of normativity, where meanings and values are mere shadows of agents’ sense-making processes or, worse, just a convenient way for us to explain their behavior.

I will claim that dispositionalist *factualism* or *realism* regarding affordances (1, above) and non-social *descriptivism* regarding life (2, above) are two instances of the same problematic approach and that we can still understand affordances relationally and dispositionally, and all forms of life in terms of a basic, non-social, sense of normativity after giving up factualism and descriptivism. Factualism and descriptivism are the metaphysical and the conceptual side of the same coin. Following the usage in contemporary expressivism, being descriptivist about an area of discourse (say, discourse about ethics or life or knowledge) is to assume that the main purpose of the discourse is to describe some feature of reality. Descriptivism goes hand in hand with factualism, according to which our normative talk is made true by independent facts and it ultimately refers to entities such as objects, properties or relations that can be scientifically described (Chrisman, 2007; Yalcin, 2011; at some points in the paper I will use “realism” instead of “factualism,” for instance when I discuss dispositionalism or moral realism, because the former term is more established in those debates). Descriptivism is closely related to representationalism, although they are not necessarily the same thesis. Antirepresentationalism can be global or local: we can claim that language in general should not be understood as aiming to represent facts or we can reject that the purpose of some specific vocabularies is to refer to entities. Local antirepresenationalism about a vocabulary amounts to antidescriptivism regarding that vocabulary (Brandom, 1994; Price, 2011). Although I sympathize with both ways to oppose representationalism, it is the local, descriptivist variety the one that I will mainly take issue with in the rest of the paper (Heras-Escribano and Pinedo García, 2018).

In order to show the ineliminability of our dispositional and normative vocabularies without acquiring dubious ontological commitments, I need to retract some of my previous statements regarding both the non-normative character of affordances and the inconsistency of having a normative take regarding non-social animals and plants (both made in conjunction with one of the editors of this issue: see Heras-Escribano et al., 2015; Heras-Escribano and Pinedo García, 2016). The strategy is to move away from the idea that we generously grant an evaluative dignity to some living creatures by finding out “facts” about them. Complex, adaptive, evolutionary behavior can only be made sense of in relational, agential and normative terms, not because of the possession of this or that inner structure (as much contemporary representationalism would have it), but because recognizing something as an agent capable of behaving is already acquiring a set of essentially normative commitments regarding what the agent *should* do and what we *should* expect from the agent, as well as what would be *better* or *worse* for the agent to find in its environment.

Assuming that there is a difference between descriptive and normative uses of language, I’d like to explore what I take to be a dilemma for cognitive science and for enactivism. On the one hand, if we apply recent debates in metaphysics to the cognitive sciences, we can say that some popular ontological approaches to affordances within ecological psychology have embraced forms of dispositional realism or factualism (Turvey, 1992). Realism regarding dispositions and, in particular, regarding affordances, has some unwelcome metaphysical consequences: in order to individuate affordances, we may need to commit to the existence of dubious entities (Tugby, 2013). In a nutshell, the difficulty is this: in order to say that an object possesses a specific affordance we cannot wait for it to manifest, because that would not account for its potential aspect and because there are affordances and dispositions that disappear when they manifest (an acorn is edible for a pig as long as it has not been already eaten, an artifact is explosive until it explodes) (for an exhaustive characterization, see Martin, 2008). What can we appeal to for their individuation? Given that we cannot individuate them in terms of their particular manifestations because, as we have said, many affordances never manifest (are never taken advantage of), we could do so in terms of their prototypical manifestation (being eaten by a pig, exploding). But we would like to say that the acorn is edible or the material is explosive even if none has been eaten or has exploded before, in which case we would need to individuate the property in terms of something which not only is a type, rather than a particular token, but which need not have ever actually manifested. Tugby (2013) defends that the best candidates are Platonic universals, i.e., universals that exist independently of whether they have ever been instantiated or not. This is hard to swallow from a naturalistic point of view [see Heras-Escribano (2017) for an exploration of a Rylean conception of disposition and Heras-Escribano (2019b) for an application to affordances; I will come back to this below].

The most obvious alternative (Chemero, 2009) is to claim that our talk of affordances has an intrinsic normative character. Here, the ecologist would be joining forces with some forms of enactivism that take normative evaluation to be adequate for any living being, whether social or not (Barandiaran et al., 2009). This other horn of the dilemma runs a two-fold risk: on the one hand, it is close to local representationalism (descriptivism) regarding normative language: even our evaluations have as their purpose referring to entities and describing facts about them. On the other, it needs to answer to the accusation of embracing a private model of rule-following (Wittgenstein, 1953; Kripke, 1982; Heras-Escribano et al., 2015; Heras-Escribano and Pinedo García, 2016): for an agent to be normatively assessed it needs to distinguish between what is correct and what it merely seems correct to it and this capacity may only be acquired by means of social sanctions and corrections. To think of affordances as intrinsic properties of things that exist independently of the agents that could perceive and take advantage of them and to think of normativity as a describable feature of the pair agent/environment may both invite the threatening thought that values are determined by independently intelligible facts (e.g., intrinsic properties of things, measurable inner forces of agents).

Of course, we could try to avoid the dilemma by reducing the demands for normative evaluation (Kiverstein and Rietveld, 2018): placing an organism within a normative network would be just a question of saying that some things were better and some worse for it. While this kind of proposal respects the distinction between describing and evaluating, it seems very hard to see in what sense it allows to distinguish between living and non-living entities. To see something as living, as an agent, as a subject of experience and behavior, is to take an ethical, rather than ontological stance, is to recognize a dignity beyond anything merely factual, as we will see in section 5. Gibsonians and neo-Gibsonians are right to insist that perception and cognition are basically active and relational. Enactivists are right to insist that to understand the living we need evaluative vocabulary. But precisely because of that, I will argue, their project should not be seen as merely discovering facts and describing processes.

## Non-Descriptivism and Rule-Following

By establishing a contrast between normativity and ontology I mean to highlight what I take to be a false dilemma that has pervaded much of the discussion regarding mind and cognition. The dilemma is this: either the mind has a causal role in nature, explainable in lawful terms, or we must sooner or later eliminate all uses of mental (cognitive, agential, intentional…) vocabulary and replace them by the vocabularies of bona fide natural sciences. If the only alternative to placing the mind causally in the world is to think of it as a mere epiphenomenon, we would seem not to have moved away too far from Descartes’ predicament. What makes the dilemma seem inescapable is a Cartesian premise shared by both horns: the only thing that we do when we speak is to refer to things (to substances, to *res*), to describe them in order to predict their behavior and control it.

So what do we mean when we say that we do different things than describing facts when we speak about the world? What else do we do other than placing entities in a nomological, spatiotemporal framework? Here is Sellars speaking about knowledge: “The essential point is that in characterizing an episode or a state as that of *knowing*, we are not giving an empirical description of that episode or state; we are placing it in the logical space of reasons, of justifying and being able to justify what one says” (Sellars, 1956, §36). What Sellars opposes is the idea that the epistemic can be analyzed in terms of non-epistemic facts, whether these facts are public or private, phenomenal or behavioral. Sellars will ultimately opt for an early version of eliminativism: given that normative spheres cannot be reduced to scientific discourse, and given that, according to him, the scientific image and the normative/manifest image compete as complete pictures of the world, we will have to eventually discard the later (Sellars, 1962).

This, however, is not the only lesson that we can take from the irreducibility of our normative vocabulary. We can also accept that we do not display the same attitude regarding the epistemic, the cognitive or even the biological than the one we take toward the merely physical. Think of Moore’s open-question argument, which he presents for ethical vocabulary but we could extend to every discourse that recognizes meanings and differential valences: someone may argue that when we say that something is good what we say is that it is pleasurable (or useful or desirable or preferred by the gods or whatever), but it will always make sense to ask “OK, it is pleasurable, but is it good?” while it makes no sense to ask “OK, it is good, but is it good?”. Even if we embraced a fully hedonistic ethics and considered that “good” refers to pleasure, we cannot conclude that both words mean the same (Moore, 1903, §13). But even someone with recalcitrant reductionist intuitions needs to recognize that different things are at issue when we disagree about something being pleasurable and when we disagree about something being good. The second disagreement is intrinsically connected with what to do, with how to live one’s life, the first isn’t (Gibbard, 2012: 42–46; Moore, 1903, § 11).

So, if we, unlike Moore, refuse to populate the world with non-natural entities such as goodness (i.e., if we insist on having a naturalistic ontology), we can still retain his conceptual non-naturalism: some of our concepts, those with normative force, are not used to refer to properties but to make intelligible the actual and potential actions of the agents that we evaluate by using those concepts (some of the most developed versions of this idea can be found in contemporary semantic expressivism; see Frápolli and Villanueva, 2012; Gibbard, 2012). Again, the idea is not that our explanatory practices themselves are subject to normative assessment, as correct, illuminating, relevant, elegant and so on, but that normativity is constitutive of the very subject matter of our explanations when it comes to agency. The lesson from these arguments is that concepts and properties are not the same: on the one hand, some concepts have a high contextual variation with respect to the properties that fall into their extension (think of “tall” or “flat”) and, on the other, there can be several concepts of the same property, some aiming at descriptively situating it within a causal network, some aiming at making salient its value for an agent. Saying that something is a better environment for a frog is different from saying that it contains lots of such and such protein, even if what makes the environment better is precisely the presence of the protein.

Let’s come back briefly to the problems of an ontological approach to affordances, one according to which affordances exist over and above the informational flows between agent and environment and the categorical properties of things. Trying to argue that affordances are intrinsic, dispositional properties of things leads to serious metaphysical difficulties, perhaps even to a commitment to Platonic universals. But this does not mean that we should abandon the idea that affordances are dispositions. We could follow Ryle’s elucidation of the explanatory power of dispositional vocabulary while, like him, avoiding the temptation of thinking that dispositions are occult forces that cause their manifestations. “There still survives the preposterous assumption that every true or false statement either asserts or denies that a mentioned object or set of objects possesses a specified attribute” (Ryle, 1949: 104; see also Heras-Escribano, 2017). Our dispositional explanations, like explanations that appeal to general laws, can be true or false not because they refer to extra entities (a causal connection, an unobservable tendency), but because they allow us to infer some factual statements from other causal statements (they are what Ryle calls inference tickets). If a thing is explosive it will explode in such and such circumstances. Ryle is particularly interested in applying this way of understanding dispositions to mental states such as beliefs in order to, among other things, reject Cartesian or representationalist understandings of the mind. My suggestion is that we fit affordances within this framework.

But, is there an evaluative, normative element involved, besides the dispositional one, when we explain behavior in terms of affordances? I think that the best way to tackle this question is in conjunction with the possibility, insisted upon by some forms of enactivism, of thinking of agency in normative terms even in the case of non-social agents. Wittgenstein (1953), §§185–243), considered by some of the most influential contemporary philosophers to be the starting point of recent focus on normativity (Kripke, 1982; McDowell, 1984; Brandom, 1994), is often interpreted as follows. If, in order to judge whether an agent has acted correctly or incorrectly according to a rule, we must interpret the rule so as to ascertain that the action is or is not an instance of what the rule calls for, then there will be no end to the chain of interpretations and reinterpretations of the rule and there will always be interpretations of the rule that take the action to be correct and others that take it to be incorrect. As long as we cannot ground our interpretation on some facts which, themselves, are interpretation-free, there is no final saying regarding rule-following.

One of Wittgenstein’s favorite examples is a child learning how to add. After some time going through examples, the teacher asks her “How much is 1000 plus 2.” The child answers “1004” and, in response to the teacher’s protestations, argues that she has done like before. “Wasn’t I supposed to add 2 extra units until 1000, 4 extra units until 10000 and so on?” This could be the beginning of an endless stubborn discussion if the pupil decided to reinterpret each word at her convenience. She may claim to understand “count” such that it works one way until 1000 and a different one after that, for instance. Can we ever stop interpreting? Wittgenstein seems to believe that we can: we can appeal to social practices, to learning, to training, to routines and customs, to being corrected by others with reinforcements and punishments of many sorts. According to the standard way of understanding Wittgenstein’s appeal to such social, public phenomena, for something to count as normative it must be placed in the context of the interaction between agents. I act correctly (regarding some norm) if I act as others do. The child in the example is making a mistake, not because she is offering the wrong interpretation of the rule, but because she is not following the socially established mathematical practices. Communities not only train us to behave as others do, but they also provide the necessary gap between it merely seeming to me that I am right and my actually being right.

If this were correct, it would make no sense to evaluate normatively the behavior of non-social agents or even the behavior of social agents when they are isolated from all communities of rule followers. The idea would be that thinking of an agent as devising and following its own rules would immediately lead to the vicious regress of interpretations: anything the agent did could fit its own rule according to some interpretation. To institute a rule and to follow it you need more than one individual (“it takes two to err”). This has been a conclusion that many, including myself, have embraced. However, now I believe this is not the only way to think of normativity nor the only way to understand Wittgenstein’s profound teachings in this sphere. Let me briefly introduce one of the usual suspects in this discussion, Robinson Crusoe. Crusoe once belonged to a (rather strict) community and he probably learned the hard way to distinguish between what merely seemed right to him from what it was considered to be right by the community. But, surely, he could still make plans and take resolutions and organize in many ways his solitary life on the island. There not being anyone around to keep him honest, could we say that he is actually following rules, that his behavior is opened to normative evaluation, his own evaluation, to start with? It seems obvious that the answer should be yes: at the very least, we could say that he has interiorized the possibility of being wrong and that would allow him to self-correct. In fact, you don’t need to be Crusoe to make up rules and to follow them. We often make decisions concerning timetables or beer consumption and manage to follow them (or fail to manage, but still realize that we are not following them) without anyone checking on us. Is the role of a community just to introduce us to the possibility of error?

There is a further, potential role the community can play and that may open up the space that we need, not for private rule-following, but for rule-following by non-social agents. The thought would be this: Wittgenstein’s target is the idea of a rule that is followed privately, that is, a rule that may only be followed by one agent. But for something to be a rule and for an action to be in accordance with it, it is sufficient that a similarly placed agent may act in a similar way to advance toward its goals. The rejection of, say, the intelligibility of a language that only I could understand (for instance, a language to refer to my sensations or my private memories) does not entail that an agent is incapable of using strategies that are fully original to itself. For this reason, I think that my previous criticism of some enactivists’ discussions of normativity with respect to bacteria and other non-social creatures was misplaced (see Heras-Escribano et al., 2015).

## Non-Social Normativity

Situated, unreflective and primitive or naive normativity are usually characterized in terms of social or communal practices or customs (see, for instance, Dreyfus, 2005; McDowell, 2007; Heras-Escribano, 2019b; Andrews, 2020). Normative considerations, whether explicit or implicit, put forward by Wittgenstein (1953) and Ryle (1949) or authors in the phenomenological tradition are linked to practice, to know-how, to bodily action but almost systematically in relation to institutions, to socially established practices. However, we could also find discussions that insist on the continuity between life and cognition where being a social creature is not a condition of possibility for the legitimate use of normative explanations (for instance, Barandiaran and Egbert, 2013; Di Paolo et al., 2017, 2018)^1^. There are two paths to the acceptance of the possibility of attributing normative features to non-social agents. One of them is to commit to the descriptivism or factualism that I am recommending against in this paper. The other is to stress the link between being a normative agent and being recognizable as such. This is the main point of this paper and it is important to explicitly avoid a certain reading of the insistence on the role of attribution, both regarding enactivism’s discussions of normativity and with respect to normative and dispositional approaches to affordances, a reading that views normativity as a useful or convenient fiction, the stance or pretense of looking at parts of the world “as if” they could act purposely and to do so better or worse. To be an agent is to evaluate the environment, to recognize opportunities, dangers and resistances, to find sense in the world and to make sense of it. To understand something as an agent is also to evaluate its behavior. But neither in the first case nor in the second the agential and normative elements are a projection from the agent to the world or from the attributor to the agent. And yet, I claim that there is a dependence between meaning and meaning-making practices and also between normativity and attributions of normativity. What kind of dependence?

Let me go back to the idea of a private model of rule-following: the possibility of an isolated agent navigating its own normative field is a powerful idea, but one that needs the background of other potential agents either acting similarly or making normative sense of its behavior. I take this to be the real revolution behind any form of antirepresentationalism, including enactivism and ecological psychology: to show the absurdity of any project that makes it intelligible to offer, from the outside, so to speak, a complete, causal description of a universe with cognition and life in it^2^. The revolution will only be complete when both faces of representationalism are discarded, the idea that our judgments are true or correct only if they correspond to one fact or another, and the idea that wherever there is cognition there must be some kind of cognitive substance or entity or organ waiting to be causally reconciled with the physical world.

The distinction between internal and external norms of evaluation can suggest that we must choose between the idea that our uses of normative vocabulary are referential and descriptive and the thought that when we evaluate behavior we project norms onto an agent which could, in principle, also be made sense of in non-normative terms. The authors of *Sensorimotor Life* make ample use of this distinction:

What is the origin of these norms? If we are not speaking of a self-individuating system, but one defined by convention, the relevant norms are also given externally to the system (…). Such would be the case of a machine designed to perform a particular purpose. What a machine “does” is thus evaluated normatively in accordance with what the designer or the user expects of it. But it is possible also to conceive of a concept of intrinsic norms (…), a concept not tied to the observer’s conventions and convenience. Intrinsic normativity cannot be the result of observers making judgments on behalf of the agent (…) (Di Paolo et al., 2017: 121; see also 102–3 and 125)

The contrast is highly intuitive. It can be at most metaphorically illuminating to take a toaster’s environment to be meaningful or dangerous for the toaster. It is bad for the toaster to suffer a sudden power surge only in the sense that the owner may have to buy a new one. Similarly, for “the thermostat knows that it is below 21 degrees Celsius” or “the printer refuses to speak to the computer,” being knowing and speaking paradigmatic normative activities. There is something unique and fundamental about entities that follow their own norms, entities for whom it matters what they find in their environment, which genuinely care about the opportunities and resistances that the world provides for them and the way they act with respect to them. The frog that fails to catch a single fly in a forest where no one can observe her dies anyway.

In this sense, I cannot but agree with the idea that “intrinsic normativity cannot be the result of observers making judgments.” And yet, the idea of intrinsic normativity, as opposed to the external norms imposed by the maker on an artifact, need not be understood in descriptivist, nomological terms. It is because the frog cares about catching flies, for her own good, that we evaluate her action as more or less suited for the task (or the environment as better or worse for her needs and goals) and not the other way around.

But to think that such caring should be explained by appeal to properties and facts about the frog is to remain within a representational view of language and to invite a backlash of reductionism and physicalism. Anyone who finds emergent properties or *possibilia* mysterious and believes that every explanatory enterprise is at bottom ontological would feel forced to pursue conceptual frugality. If all our concepts aim at referring to entities but there are only physical entities, then we will have to eliminate most of our concepts (see Pinedo García, 2016). In contrast, we can accept the existence of a plurality of ineliminable explanatory approaches, some mechanistic, some agential, intentional and normative (see Pinedo García and Noble, 2008).

## Ontology and Ethics

The worry I have been trying to express is that a descriptivist, ontological reading of our explanations of agency in terms of affordances and in terms of intrinsic norms may obscure the evaluative dimension of such explanatory practices and, perhaps, invite reductionist and eliminativist agendas that try to do without the idea of a meaningful environment or a normative encounter with it. Part of my concern has to do with what I have called a residual representationalism regarding language. My insistence on the fact that there is some inescapable ethical and normative aspect in our approach to the living is shared by many enactivists and ecological psychologists. For instance, the authors of *Linguistic Bodies* are quite clear about the ethical dimension related to meaning and intentionality, even in their most basic forms:

(…) there is an ethical dimension (…) entailed by our theory (…) [I]f living organisms are autonomous sense-makers that behave in relation to vital norms, this implies that they are recipients of ethical concern. This must be reflected and not occluded by the language we use to talk about them (Di Paolo et al., 2018: 34).

Ethical concern is not something that is added to already constituted linguistic bodies, as sociocultural normativity is supposedly added to a presumed original nature in dualistic thought (Ibid.: 310).

Perhaps an unavoidable, though unfortunate, consequence of the still felt Cartesian influence is a tendency in the philosophy of the cognitive sciences to play the “you are more dualist than I am” game. But it may help to pause and see whether the game is always played with the same rules or whether there is some basic equivocation in the appeal to dualism. Unlike eliminativism, which is better understood as a linguistic than as an ontological thesis, being a dualist involves an ontological commitment with the existence of things of radically different nature, in the extreme case, so different that it is a mystery how they could causally relate to one another. In contrast, to affirm that the language of, say, art criticism or gastronomy cannot be replaced by the language of molecular biology should not be seen as a dualistic statement. So, inasmuch as dualism concerns what there is and not our ways of making sense of it, dualistic thought, at a minimum, involves taking norms, meanings or values to be entities and that’s where the problems start. However, one can accept, wholeheartedly and for normative and ethical reasons, the need to understand living being’s interactions with their environment as meaningful or significant without feeling the urge to populate the world with meanings and values. We would be avoiding dualism at the price of embracing a false dilemma: in one horn we would have values and norms out there, either waiting to be found or merely projected by the living, in the other, they would be just a manner of speaking, a stance we can choose to take toward nature.

Both poles of this dilemma share a problematic premise: the purpose of our explanatory vocabularies is to represent facts, so either there are facts about meaning and value or our normative practices are provisional shortcuts, ready to be discarded as our knowledge develops. But factualism, interpretativism and eliminativism can be all avoided if we recognize that there is a lot more that we do with our words than representing, predicting and controlling nature. This is where the best analytic philosophy of language, from the early days, can still open up a space that contemporary debates often ignore: Moore, Wittgenstein, Ryle or Austin (who coined the expression “descriptive fallacy”, see Austin, 1962/1979) all share this antidescriptivist approach and it is depressing that so much contemporary philosophy of mind seems to stem directly from ways of thinking that were deeply challenged by these philosophers. My main purpose in this paper has been to question that to oppose functionalism in the philosophy of mind and cognitivism in psychology we should share their ontological playfield and quarrel about what there is. Our mundane values or our free-standing dispositional affordances may be preferable to their representations and computations, but we will be turning a debate that should focus on the normative and ethical dignity of the living on a Cartesian debate about measurable substances and attributes.

One of the consequences of this Cartesian heritage is the disproportionate importance that the mind-body problem has had in the philosophy of mind, especially in the analytic tradition, but not exclusively. The mind-body problem is an ontological problem and the different solutions that have been proposed are descriptive: they try to establish what kind of entity a mind is in order to account for its causal interaction with the physical world. But this concentration on the mind-body distinction has tended to hide a more important one, the distinction between an agent and a thing, a distinction which is not so much descriptive or ontological as normative or ethical (see Ramberg, 2000; Rorty, 2000). In his debate with Rorty, aptly situated under the heading “post-ontological philosophy of mind,” Ramberg insists on this point and attributes to Davidson a poignant form of subversiveness against the philosophy of mind mainstream. The same way that I have extended Wittgenstein’s thoughts on normativity beyond language and society, I’d like to apply Ramberg’s point to all forms of agency:

[T]he vocabulary of agency leaves us better off, better in the sense of “politically more free.” I see Davidson as providing a tool, a marginal tool (…) in a struggle against the steady spread of dehumanizing, homogenizing management of human existence that is the real threat of scientism. Scientism is not bad, I am sure Rorty would agree, because it gets the world wrong, or even because it is a rehash of Kantian and Platonic ontology, but because it renders us subject to certain forms of oppression (Ramberg, 2000: 367).

The ethical concern brought about by agency-vocabulary is an obstacle to treating living creatures, and us humans, as ownable and controllable entities. But there is another desideratum, besides avoiding a view of agents as mere things: not making everything into an agent. Although our resistance to accepting a normative treatment of non-social creatures (Heras-Escribano et al., 2015) is now, to my eyes, misplaced (if we think that using normative vocabulary is adopting a perspective that transcends mere description and prediction and, hence, is not in the business of following track to intrinsic properties of agents), it was an overreaction to a genuine worry: the risk that we will end up extending our normative nets to spheres where they are not needed. In the past, we have expressed this worry by pointing out that naturalizing normativity cannot be achieved by normativizing nature on pains of a dangerous form of idealism (Ibid.). To embrace the thought that there are areas beyond human linguistic and institutional practices that should be understood normatively should not make us think that there are no important distinctions to be made, however, fuzzy the borders may be, between prediction and control and understanding and ethical concern. One of the strategies of oppressive power is to present issues of value as factual and factual questions as matters of opinion. The former can be found in the attempt to put in technocratic hands decisions regarding public policies (cf. Samuel Huntington’s advice to the trilateral commission: “to employ the language of expertise more widely as a mechanism to deal with the ‘excesses of democracy,”’ Stanley, 2015: 210)^3^. and the latter in the efforts to muddle scientific consensus with allegedly discording voices in cases such as climate change or the connection between tobacco smoking and certain types of cancer. If we suggest that everything is normative, some powerful tools of political resistance may be lost.

I have assumed from the beginning that enactivism, ecological psychology and the non-descriptivist and antirepresentationalist strands of analytic philosophy share a commitment with the need to start with the vocabulary of agency, of meaning and value, if we are to do justice to life and cognition. But I believe that what gives philosophical and ethical edge to this commitment may be lost if we present ourselves as pursuing a project that competes with standard, non-normative forms of approaching nature, as merely offering richer redescriptions of a world that others may try to describe with the vocabulary of the physical sciences. The reduction of living beings to things, of value to price, of the sphere of normative negotiation amongst legitimate options regarding how to live to a technocratic calculus of benefits, are all equally threatening consequences of blurring the distinction between evaluation and description.

## The Inescapability of the Normative Dimension

All attempts at explaining or understanding any kind of phenomena have a normative nature. We can have better and worse explanations, our understanding can be deeper or shallower. Our explanations can be wrong and there is always room for misunderstanding, whether we are dealing with subatomic particles or with cultural practices. This is the simplest argument in favor of the irreducibility and ineliminability of our normative vocabulary: even the most purist reductionist and eliminativist projects need to be evaluated. However, sometimes the very subject matter of our explanations seems to need tackling by using normative vocabulary. Is this just a convenient stance, a provisional perspective that we adopt when we lack the knowledge or the time needed to give a causal explanation, grounded on laws rather than on norms? There are strong reasons to doubt this. One of them is a consequence of the point I have just highlighted regarding *all* of our explanatory practices: at the very least, when we are interested in understanding nature our activities are guided by norms, norms of coherence, of simplicity, of empirical adequacy, of truth…But if some of our behavior necessarily has to be seen as an instance of rules being followed better or worse, correctly or incorrectly, the very idea that normativity is a non-compulsory stance loses its strength and the possibility, dear to some enactivists and ecological psychologists, that acting, perceiving, in sum, being alive, is to be normatively regulated becomes more attractive.

Making sense of agency in terms of rules and norms, of evaluations, of relationships of meaning and relevance, of opportunities and dangers being properly or wrongly tackled, cannot be a question of projecting a certain explanatory frame into nature on pain of devoiding agents of their constitutive normativity and reducing them to mere things, objects to be controlled and possessed. But we should also avoid the opposite temptation, the temptation of thinking of an agent as a mechanism whose wheels should find the right preexisting rails, as if sense, meaning and correctness were things that could be understood in descriptive and nomological terms independently of agents’ practices of making sense of the world. The danger of putting too much emphasis on ontological matters is to lose sight of what I have placed on the side of the ethical and normative: that the intrinsically ethical dignity linked with being an agent is not something we generously bestow on the living, but it is not something we find in there by merely aiming at predicting nature in order to control it (McDowell, 1984; Ramberg, 2000). The problem that lurks behind the rails metaphor is that, besides the danger of giving pride of place to an understanding of the world, including the biological world, as something to be predicted, controlled and, ultimately, owned by us, there is a different, although related, menace behind the descriptivism I have been warning against: to think that we are in the business of describing normative facts when we approach agency could give us a sense of entitlement to treat agents as mere things if we fail to discover such facts. Hartry Field puts the point forcefully concerning moral realism, but it can be extended to any version of factualism regarding the normative:

Why make our policies conditional on our beliefs about the existence and nature of normative facts? If we morally disapprove of torturing dogs, why rest this disapproval on a pure belief that there is a straightforward normative fact that we oughtn’t torture dogs? Indeed, *I’m tempted to say that the moral realist has not only a dubious metaphysics, but also a dubious morality* that allows torturing dogs under the condition that there are no straightforward moral facts, or under the condition that those moral facts permit or even require such torture (Field, 2009: 270, my italics).

The temptation which I fear concentration on ontological matters brings is this: to avoid a subjectivist projectionism according to which normativity is just a manner of speaking, we want to say that agents follow their own norms independently of whether we understand their behavior in terms of such norms or not. But this should not lead us to think of criteria of correctness and norms as transcending the contingent activities and practices, either solitary or public, of agents. My insistence on the ethical aspect of our normative vocabularies is a way of highlighting the difference between measuring the radioactive decay of a group of atoms and explaining how plants communicate. We need to engage in meaningful and communicative interactions to understand plant communication, but we don’t need to be radioactive to explain the behavior of Uranium-238.

Our distinction between the animate and the inanimate world is, at the end of the day, a distinction between having an internal perspective and having an external perspective on things (or, rather, between things that demand an internal perspective and things that do not). Even practices that are fully alien to us, both culturally and biologically, can only be understood if we grasp what is it that the practitioners value, why do they care, what’s at issue for them, how can they satisfy better or worse their needs or preferences. In sum, why it makes sense for them to live their life that way. Taking an internal perspective cannot amount to sharing a way of living, at least not in most cases. But it must involve recognizing it as a way of living and that implies going beyond the ontological and into the ethical.

## Conclusion

In this paper I have tried to offer an alternative to the tendency, sometimes implicit, to think of normativity in descriptive, ontological terms. To do so, I have brought back into contemporary post-cognitivist debates some central ideas from early analytic philosophy because I’m convinced that they target forms of representationalism that have often remained invisible and that have led to otherwise escapable dilemmas resulting from a confusion between different explanatory projects. I have pointed out as problematic some forms of descriptivism, which I have identified as a form of local representationalism, and claimed that blurring the distinction between placing something within a causal, factual network and evaluating it or, in other words, mixing up ontological and ethical matters, opens up the door for an oppressive and objectivizing view of nature, one according to which agents can be treated as mere things, subject to prediction, control and ownership. To make normative, evaluative matters depend on factual discoveries can lead to inaction in situations that demand moral engagement.

## Author Contributions

The author confirms being the sole contributor of this work and has approved it for publication.

## Conflict of Interest

The authors declare that the research was conducted in the absence of any commercial or financial relationships that could be construed as a potential conflict of interest.

## Footnotes

1 An obvious exception is radical enactivism, a position that is informed by the early analytic ideas presented in this paper and which mostly avoids approaching agency and cognition from a descriptive and ontological perspective (see Hutto and Myin, 2012).

2 I’m thankful to Neftalí Villanueva for pressing me on several points, including this one.

3 “Al Smith once remarked that “the only cure for the evils of democracy is more democracy.” Our analysis suggests that applying that cure at the present time could well be adding fuel to the flames. Instead, some of the problems of governance in the United States today stem from an excess of democracy (…). Needed, instead, is a greater degree of moderation in democracy. (…) First, democracy is only one way of constituting authority (…). In many situations the claims of expertise, seniority, experience, and special talents may override the claims of democracy as a way of constituting authority” (Crozier et al., 1975: 113).
